# Grapes

**Published:** 1874-10

**Authors:** 


### GRAPES.

Half a pound or more of grapes, taken as a "dessert," half an hour before the two or three regular meals of the day, with nothing whatever between, will, in a few days, remove a great variety of symptoms, those for example which result from

BILIOUSNESS, such as bad taste in the mouth of morning, no special relish for food, aversion to meats, sleepiness, indisposition to exercise, indifference, dullness, thick or bitter tongue, unusual thirst, restlessness, headache, cold feet, chilliness, confined condition of the system, depression of spirits, all comprised in the expression,

"I FEEL MISERABLE."

A bilious person does not have all these symptoms at once, but several of them. The grapes, by their agreeable acidity, so act on the system as to relieve it of its bile, and thus remove the cause of the symptoms enumerated, and that is, "cure." The immediate cause of all the discomfort is a "confined" condition of the system; the seeds of the grapes act as an irritant as they pass along the alimentary canal and cause it to "water," just as the eye "waters" if a hard substance touches it; this watering dissolves the more solid matters contained in the intestines, "washes" them out, and the man is well. The covering of the grapes should be chewed, but not swallowed.

By the same means an innumerable train of dyspeptic symptoms, too numerous to mention, may be modified, and many times removed, if the treatment is followed up persistently. If any one is conscious of being cross, fretful, peevish, complaining, ill-natured, finding fault with everybody and everything, never satisfied, always "blue," always morose, growling if you do, and growling if you don't; beginning on opening the eyes in the morning, and only ending with the last word at night, such a person has

DYSPEPSIA, making himself miserable, and wounding and distressing everybody who has anything to do with him; a terror to the family if a man, and a misery to the whole household, if a woman. Of many such, when they at last die, it is felt, if not expressed, "a good riddance, anyhow." And lo! these are only a part of her ways, only a part of the "shines" which the miserable dyspeptic "cuts." It is almost a pity that such people can be cured by so pleasant a remedy as eating grapes. But they can be in cases not a few, especially if supplemented by an hour's leisure walk in the open air, in the fore and afternoons.

If a medicine was brought to the attention of every sedentary person, those living mostly in doors, who are either dyspeptic or bilious, a hundred thousand dollars worth of it would be sold in a year, especially if the constituents were not known, and they were discovered accidentally by some old crone who had been living in a mud-hut, "all done by herself," in some almost inaccessible forest. But inasmuch as the same thing can be done by the simple means of eating grapes, costing ten cents, or nothing, a pound, it is doubtful if two readers out of a hundred will give the remedy a fair trial. May be, if the reason of the thing is given, it may have a better effect. Every dyspeptic has brought on his calamities by having made a pig—no, not a pig of himself, for pigs have more sense—but a fool of himself, by eating too much; by gormandizing at every meal. Almost always dyspeptics have a good appetite, an appetite for more than can be digested; hence, although they eat a great deal, nature has not the power to draw from the food the nourishment which it contains, hence the dyspeptic is always hungry, only not miserable, while he is eating, and in ten minutes thereafter his tortures begin anew, to last, in many cases, for hours, as the mass of food, not being digested, lays like a load in the stomach. The grapes take away the sharpness of the appetite for the regular meal, hence not half as much boiled food is eaten.

